# Polysulfides (H_2_S_n_) produced from the interaction of hydrogen sulfide (H_2_S) and nitric oxide (NO) activate TRPA1 channels

**DOI:** 10.1038/srep45995

**Published:** 2017-04-05

**Authors:** Ryo Miyamoto, Shin Koike, Yoko Takano, Norihiro Shibuya, Yuka Kimura, Kenjiro Hanaoka, Yasuteru Urano, Yuki Ogasawara, Hideo Kimura

**Affiliations:** 1 Department of Molecular Pharmacology, National Institute of Neuroscience, National Center of Neurology and Psychiatry, 4-1-1 Ogawahigashi, Kodaira, Tokyo 187-8502, Japan; 2 Department of Analytical Biochemistry, Meiji Pharmaceutical University, 2-552-1 Noshio, Kiyose, Tokyo 204-8588, Japan; 3 Graduate School of Pharmaceutical Sciences, The University of Tokyo, 7-3-1 Hongo, Bunkyo-ku, Tokyo 113-0033, Japan

## Abstract

Hydrogen sulfide (H_2_S) exerts synergistic effects with another gaseous signaling molecule nitric oxide (NO) on ion channels and vasculature. However, the mechanism of the synergy is not well understood. Here, we show that the interaction between H_2_S and NO generates polysulfides (H_2_S_n_), which activate transient receptor potential ankyrin 1 (TRPA1) channels. High performance liquid chromatography with tandem mass spectrometry analysis, along with the imaging of intracellular Ca^2+^ and H_2_S_n_, showed that H_2_S_n_ and their effects were abolished by cyanolysis and by reducing substances such as dithiothreitol (DTT), cysteine, and glutathione (GSH). However, the effects of nitroxyl or nitrosopersulfide, other potential products of H_2_S and NO interaction, are not affected by cyanolysis or reducing substances. This study demonstrates that H_2_S_n_ are products of synergy between H_2_S and NO and provides a new insight into the signaling mechanisms.

Hydrogen sulfide (H_2_S) has various physiological roles: neuromodulation, vascular tone regulation, cytoprotection against oxidative stress or ischemia reperfusion injury, and anti-inflammation^1^,^2^,^3^,^4^,^5^,^6^. Cross talk between H_2_S and another signaling molecule, nitric oxide (NO), was initially reported as a synergistic effect of relaxation on vascular smooth muscle^2^. A similar synergistic effect of both molecules was observed in the twitch responses of the ileum^7^.

Two mechanisms of H_2_S and NO interaction were proposed for angiogenesis: the effect of H_2_S is mediated by NO through the activation of endothelial NO synthetase (eNOS) in one mechanism^8^, while the cooperative action between H_2_S and NO is essential in another^9^. Increase in H_2_S production and expression of an H_2_S-producing enzyme, cystathionine γ–lyase (CSE), by NO were also reported^3^. H_2_S enhances the activity of eNOS by facilitating phosphorylation of an active site and de-phosphorylation of the inhibitory site to increase the production of NO, leading to the attenuation of sudden cardiac arrest-induced mitochondrial injury, as well as protection of the heart from ischemia-reperfusion injury^10^,^11^.

It has been proposed that molecules generated by the chemical interaction of H_2_S and NO show a greater activity than the parental molecules or play a role as their carrier^12^,^13^,^14^,^15^. Nitroxyl (HNO) generated by the interaction of H_2_S and NO was proposed to activate transient receptor potential ankyrin 1 (TRPA1) channels^14^. However, nitrosopersulfide (SSNO) was reported to be mainly generated from H_2_S and NO interaction to act as a NO carrier, releasing NO to relax vascular smooth muscles^15^. Although H_2_S_n_ were detected as additional common products in both studies, they have not been studied in detail^14^,^15^,^16^. The application of H_2_S- or NO-donor alone can produce H_2_S_n_ probably due to the interaction with endogenous H_2_S or NO in mast cells^17^.

Recently, we found that H_2_S_3_ and H_2_S_2_ are produced by 3-mercaptopyruvate sulfurtransferase (3MST), an H_2_S-producing enzyme, from 3-mercaptopyruvate (3MP), as well as by the oxidation of H_2_S^18^,^19^,^20^. In addition, H_2_S_n_ activate TRPA1 channels by sulfurating two cysteine residues at the amino-termini of the channels^21^,^22^,^23^,^24^. Various other effects of H_2_S_n_ were subsequently reported. H_2_S_n_ facilitates the translocation of nuclear factor-like 2 (Nrf2) to the nucleus by modifying its binding partner, kelch-like ECH-associated protein 1 (Keap1), to up-regulate the transcription of antioxidant genes^25^. It also regulates the activity of a tumor suppressor, phosphatase and tensin homolog (PTEN)^26^, activates protein kinase G1α to relax vascular smooth muscle^27^, and suppresses the activity of glyceraldehyde 3-phosphate dehydrogenase (GAPDH)^28^.

The present study showed that H_2_S_2_ and H_2_S_3_ were generated by the chemical interaction of H_2_S and NO. H_2_S_n_ and their effects on TRPA1 channels were abolished by cyanolysis and by reducing substances, such as dithiothreitol (DTT), cysteine, and glutathione (GSH). However, HNO is resistant to cyanolysis, and SSNO^−^ is resistant to reducing substances. These observations suggest that H_2_S_n_, rather than HNO or SSNO^−^, are involved in the activation of TRPA1 channels.

## Results

### Generation of H_2_S_2_ and H_2_S_3_ by the chemical interaction of H_2_S and NO

**Figure 1:**
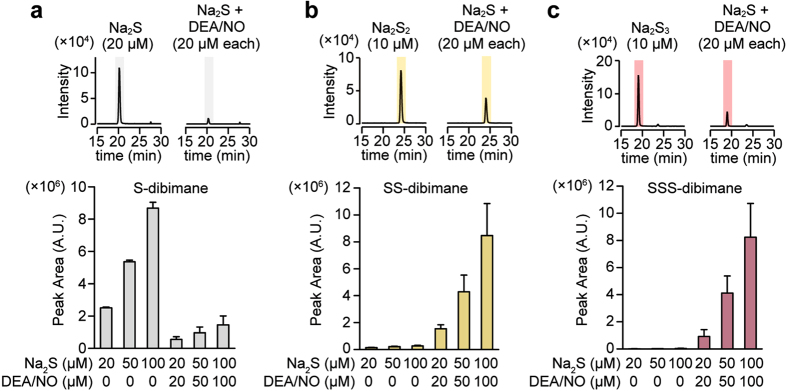
LC/MS/MS analysis of products generated by the interaction of H_2_S with NO. (**a**,**b**,**c**) Chromatograms and relative levels of dibimane-derivatives of H_2_S (S-dibimane) (**a**), H_2_S_2_ (SS-dibimane) (**b**), and H_2_S_3_ (SSS-dibimane) (**c**) generated in the mixture of Na_2_S and DEA/NO. (n = 3).

The oxidation of H_2_S generates H_2_S_n_^20^,^21^,^22^,^23^,^29^, and the interaction of H_2_S with S-nitroso cysteine generates cysteine persulfide^30^,^31^. Therefore, it is possible that the interaction of H_2_S with NO produces H_2_S_n_. This possibility was examined using high performance liquid chromatography with tandem mass spectrometry analysis (LC-MS/MS). A mixture of Na_2_S, a sodium salt of sulfide, and diethylamine NONOate (DEA/NO), a donor of NO, was derivatized with monobromobimane, a fluorescence dye specific to thiols, and analyzed using LC-MS/MS. H_2_S_2_ and H_2_S_3_ were generated by consuming H_2_S after mixing Na_2_S and DEA/NO in a concentration dependent manner (Fig. 1).

### Activation of TRPA1 channels by H_2_S_2_ and H_2_S_3_ generated by the interaction of H_2_S with NO

**Figure 2:**
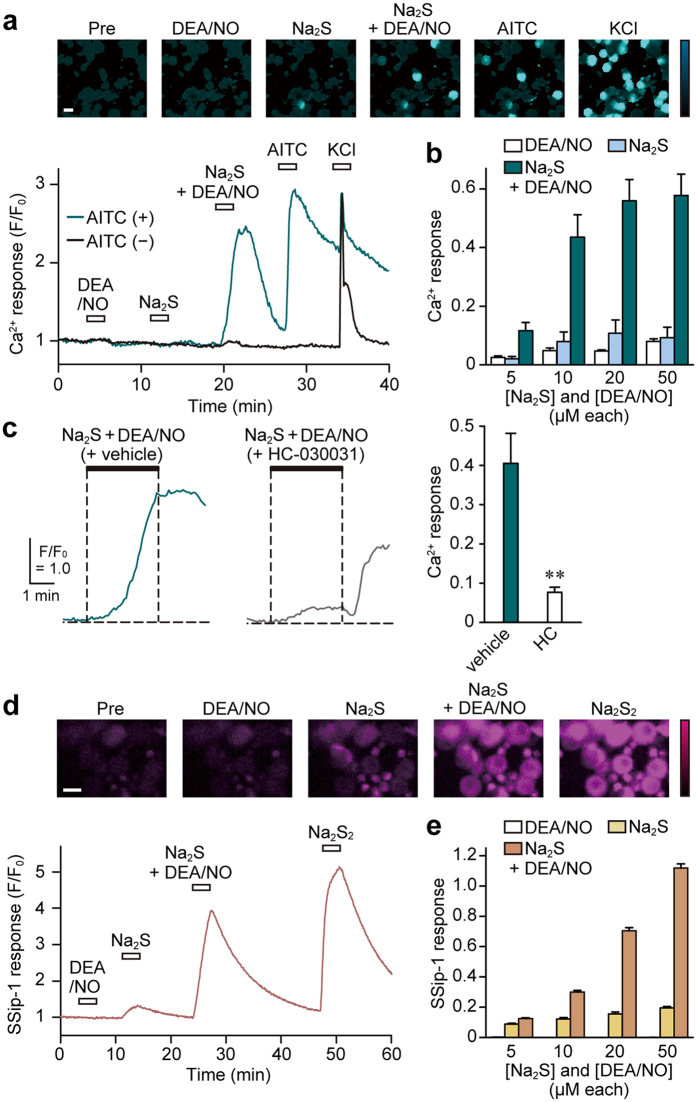
Generation of H_2_S_2_ and H_2_S_3_ by the interaction of H_2_S with NO, and their effect on TRPA1 channels. (**a**) Ca^2+^ influx induced by the product of H_2_S and NO interaction. The images and traces of Ca^2+^ influx induced by 20 μM Na_2_S, 20 μM DEA/NO, a mixture of 20 μM each of Na_2_S-DEA/NO, 100 μM AITC, and 50 mM KCl detected in fluo-4-loaded AITC-responsive and -unresponsive DRG neurons are shown. The bar in the image indicates 30 μm. (**b**) Concentration-Ca^2+^ response relations for H_2_S_n_ generated from H_2_S and NO. Data were obtained from AITC-responsive cells (n = 29–37). Ca^2+^-responses were normalized to those obtained with 50 mM KCl applied for 5 min after the application of AITC. (**c**) Ca^2+^ influx induced on the activation of TRPA1 channels by the products generated by the interaction of H_2_S with NO. The products from Na_2_S and DEA/NO induced Ca^2+^ influx that was suppressed by TRPA1 channel specific inhibitor, 30 μM HC-030031, in DRG neurons pre-loaded with fluo-4. (n = 30–33). (**d**) The intracellular levels of H_2_S_n_ in cells applied with H_2_S and NO. Responses to 20 μM Na_2_S, 20 μM DEA/NO, a mixture of 20 μM each of Na_2_S-DEA/NO, and 30 μM Na_2_S_2_ are shown in DRG neurons pre-loaded with SSip-1. (**e**) Concentration-SSip-1 response relations for H_2_S_n_ generated from H_2_S and NO (n = 28–30). SSip-1 responses were normalized by those to 30 μM Na_2_S_2_, applied 20 min after exposure to Na_2_S-DEA/NO mixture. The bar in the image indicates 30 μm. Data are represented as means ± SEM. **p < 0.01 (unpaired Student’s *t*-test).

H_2_S_n_ activates TRPA1 channels in astrocytes and dorsal root ganglion (DRG) neurons^23^,^24^. These observations and above-mentioned results suggest the possibility that the mixture of H_2_S and NO activates TRPA1 channels through the generation of H_2_S_2_ and H_2_S_3_. The activation of TRPA1 channels in DRG neurons by mixture of H_2_S and NO was examined by measuring the Ca^2+^ influx with Fluo-4, a Ca^2+^ sensitive fluorescence dye. The mixture of Na_2_S and DEA/NO induced Ca^2+^ influx in DRG neurons, which were sensitive to allyl isothiocyanate (AITC), an agonist of TRPA1 channels, and suppressed by HC-030031, an antagonist of the channels, in a concentration-dependent manner. However, Na_2_S or DEA/NO alone induced negligible response (Fig. 2a,b and c; Supplemental Fig. 1a and b). These observations suggest that the interaction of H_2_S with NO generates H_2_S_2_ and H_2_S_3_ that activate TRPA1 channels.

H_2_S_2_ and H_2_S_3_ generated by the reaction of H_2_S and NO in DRG neurons were also examined using SSip-1, a fluorescence probe that specifically and reversibly binds to sulfane sulfur^32^ (Supplemental Fig. 2). The levels of H_2_S_n_ generated by a mixture of Na_2_S and DEA/NO increased in a concentration-dependent manner in DRG neurons, while Na_2_S alone resulted in a slight increase in H_2_S_n_ levels, and no changes were induced by DEA/NO alone (Fig. 2d and e). HNO and H_2_O_2_, which activate TRPA1 channels, were not detected by SSip-1^14^,^33^ (Supplemental Fig. 3a and b). These observations suggest that the interaction of H_2_S with NO produces H_2_S_2_ and H_2_S_3_ that activate TRPA1 channels.

### Instantaneous generation of H_2_S_n_ on exposure of H_2_S to NO

**Figure 3:**
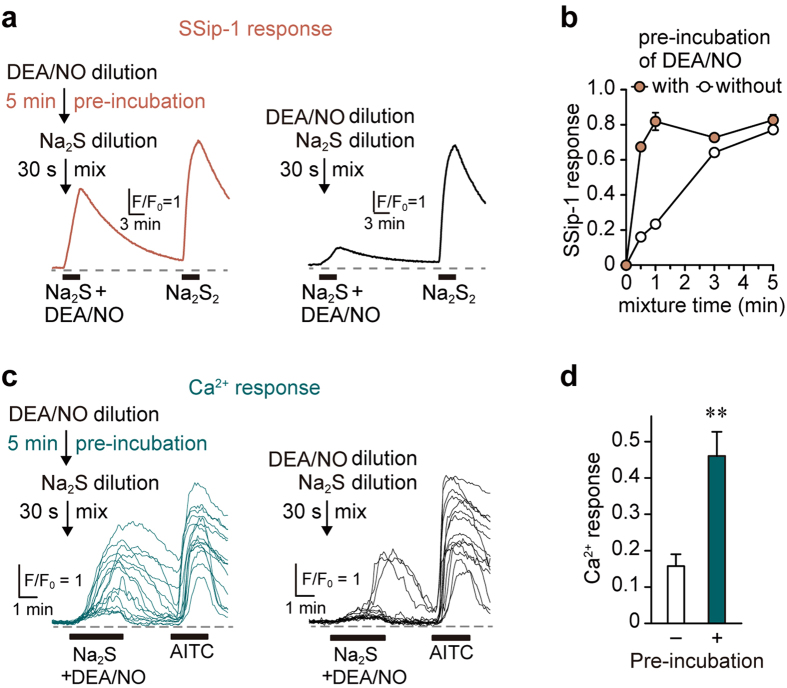
H_2_S_n_ is produced immediately after the exposure of H_2_S to NO. (**a**) A release of NO from DEA/NO is rate-limiting for the generation of H_2_S_n_. SSip-1 responses to Na_2_S-DEA/NO mixtures with (w) or without (w/o) 5 min incubation of DEA/NO in HBS, before mixing with Na_2_S, are shown. (**b**) The time-course of the production of H_2_S_n_ after mixing Na_2_S and DEA/NO with or without 5 min pre-incubation (n = 29–30). SSip-1 responses were normalized by those obtained on exposure to 30 μM Na_2_S_2_. (**c**) Ca^2+^ influx induced by H_2_S_n_ produced by the mixture of H_2_S with NO. Fifteen traces are shown. Twenty micro molar Na_2_S was mixed for 30 sec with 20 μM DEA/NO with or without 5 min-pre-incubation in HBS. (**d**) The amplitude of Ca^2+^ influx induced by the product of H_2_S and NO depends on the pre-incubation of DEA/NO to release NO. Release of NO from DEA/NO rate-limits the generation of H_2_S_n_ and the induction of Ca^2+^ influx in AITC-responsive cells (n = 25–28). Data are represented as means ± SEM. **P < 0.01 (unpaired Student’s *t*-test).

The rate of H_2_S_n_ generation from H_2_S and NO was examined. Na_2_S releases H_2_S immediately after dissolving in the medium, while DEA/NO slowly releases NO after its dissolution^34^. Therefore, it is possible that the generation of H_2_S_n_ depends on the release of NO into the medium. The levels of H_2_S_n_ generated from the mixture of Na_2_S and DEA/NO were measured by SSip1 with or without pre-incubation with DEA/NO. The Ca^2+^ influx induced by the products from Na_2_S and DEA/NO was also examined. Thirty seconds after mixing Na_2_S with DEA/NO solution, which had been dissolved for 5 min, NO was fully released from DEA/NO and generated H_2_S_n_ (Fig. 3a and b) that effectively induced Ca^2+^ influx (Fig. 3c and d). However, the mixture of Na_2_S and DEA/NO without any pre-release of NO generated much less amount of H_2_S_n_ after 30 seconds (Fig. 3a and b) and induced a weak Ca^2+^ influx (Fig. 3c and d). The release of NO from DEA/NO is a rate-limiting step for the generation of H_2_S_n_. Once NO is released into the reaction mixture, H_2_S_n_ is immediately produced (Fig. 3b). Incubation of Na_2_S with DEA/NO fully for 5 min was thus used for the other experiments in this study, as it fully generates H_2_S_n_.

### H_2_S_n_ generated from H_2_S and NO was degraded by cyanolysis but not HNO

**Figure 4:**
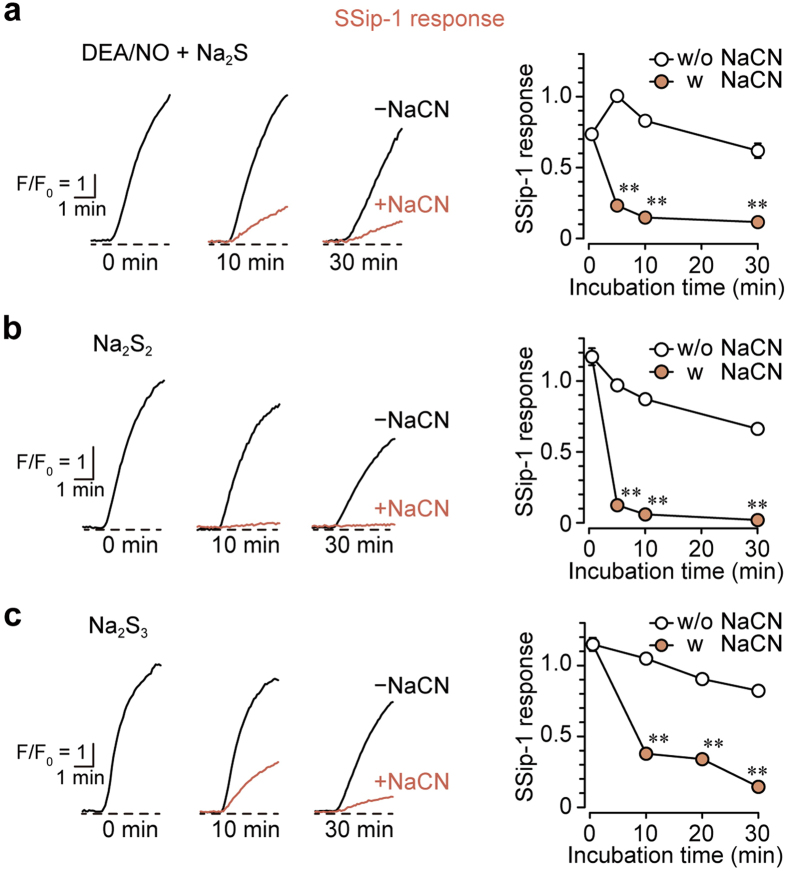
The reaction products of H_2_S and NO are degraded by cyanolysis. (**a**,**b** and **c**) The products of H_2_S and NO (**a**), H_2_S_2_ (**b**) and H_2_S_3_ (**c**) were degraded by cyanolysis. Mixtures of 20 μM each of Na_2_S and DEA/NO after incubation for 5 min, and 10 μM each of Na_2_S_2_ and Na_2_S_3_ were treated with 20 mM NaCN. After incubation with NaCN or NaCl as a control, each solution was applied to SSip-1-loaded DRG neurons (n = 29–30). SSip-1 responses were normalized with those obtained on exposure to 30 μM Na_2_S_2_.

It has been proposed that H_2_S interacts with NO to produce HNO, which activates TRPA1 channels^14^. HNO is resistant, while H_2_S_n_ is sensitive, to cyanolysis^14^,^35^. We examined sensitivity of the product of H_2_S and NO interaction to cyanolysis using SSip-1 in DRG neurons. The mixture of Na_2_S and DEA/NO, which was incubated for 5 min to fully produce H_2_S_n_, was exposed to NaCN, and the mixture was applied to DRG neurons. The exposure to NaCN dramatically decreased the amount of H_2_S_n_ generated from H_2_S and NO (Fig. 4a). A similar vulnerability to cyanolysis was observed for H_2_S_2_ and H_2_S_3_ (Fig. 4b and c; Supplemental Fig. 3c and d). These observations suggest that molecules generated by the interaction of H_2_S with NO are H_2_S_2_ and H_2_S_3_, rather than HNO.

**Figure 5:**
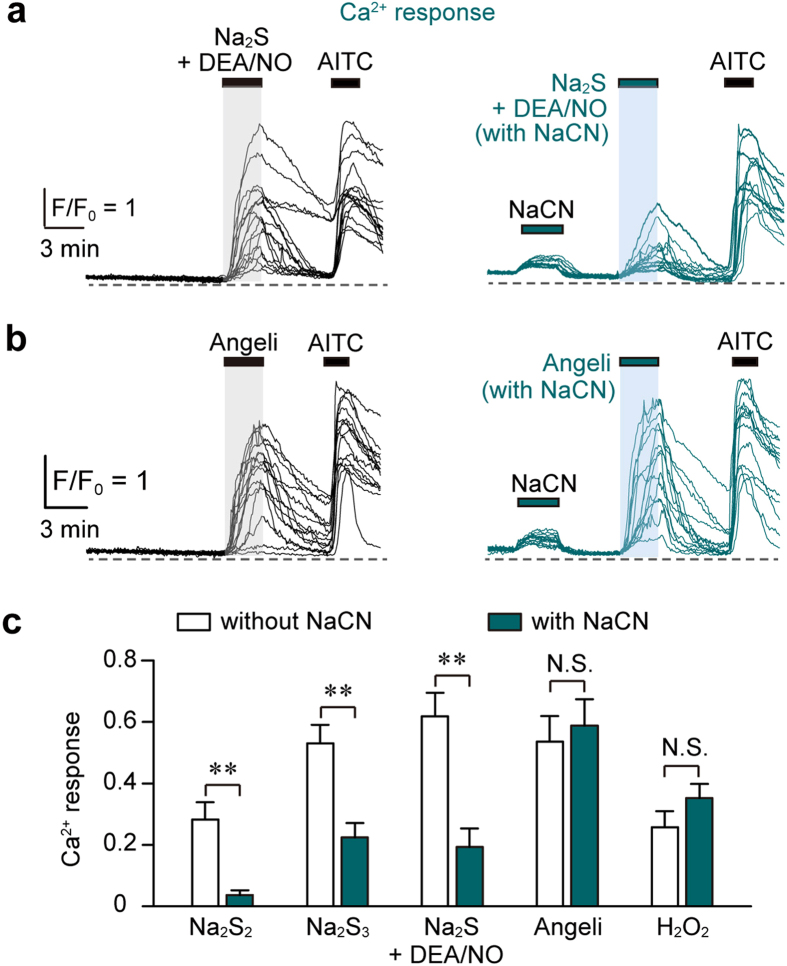
Ca^2+^ influx induced by the mixture of H_2_S and NO is abolished by cyanolysis, but not that induced by HNO. (**a**,**b**) Ca^2+^ responses were induced by the mixture of 20 μM each of Na_2_S and DEA/NO (**a**) or 0.3 mM Angeli’s salt (**b**) pre-incubated for 10 min in the presence or absence of 20 mM NaCN. (**c**) Sensitivity of Ca^2+^ influx induced by the interaction of H_2_S with NO, as well as HNO, to cyanolysis. Ca^2+^ responses induced by 10 μM Na_2_S_2_, 10 μM Na_2_S_3_, a mixture of 20 μM each Na_2_S and DEA/NO, 0.3 mM Angeli’s salt, and 1 mM H_2_O_2_ in the presence or absence of 20 mM NaCN are shown. Data were obtained from AITC-responsive cells (n = 26–33). Angeli’s salt and H_2_O_2_ were treated with NaCN immediately after dilution in HBS. Ca^2+^-responses were normalized by those obtained in response to 50 mM KCl. Ca^2+^-responses non-specifically induced by NaCN were subtracted from those induced by each stimulant in the presence of NaCN. Data are represented as means ± SEM. **P < 0.01 (unpaired Student’s *t*-test).

We confirmed these results by measuring Ca^2+^ influx induced by the mixture of H_2_S with NO in DRG neurons. The Ca^2+^ influx induced by the mixture of Na_2_S and DEA/NO, as well as that by Na_2_S_2_ and Na_2_S_3_, was suppressed by cyanolysis, while that induced by Angeli’s salt, a HNO donor, and H_2_O_2_ was not affected by cyanolysis (Fig. 5). These observations confirmed that H_2_S_2_ and H_2_S_3_, but not HNO, were generated from H_2_S and NO to activate TRPA1 channels.

### H_2_S_n_ generated by the interaction of H_2_S and NO was degraded by reducing substances but not SSNO^−^

**Figure 6:**
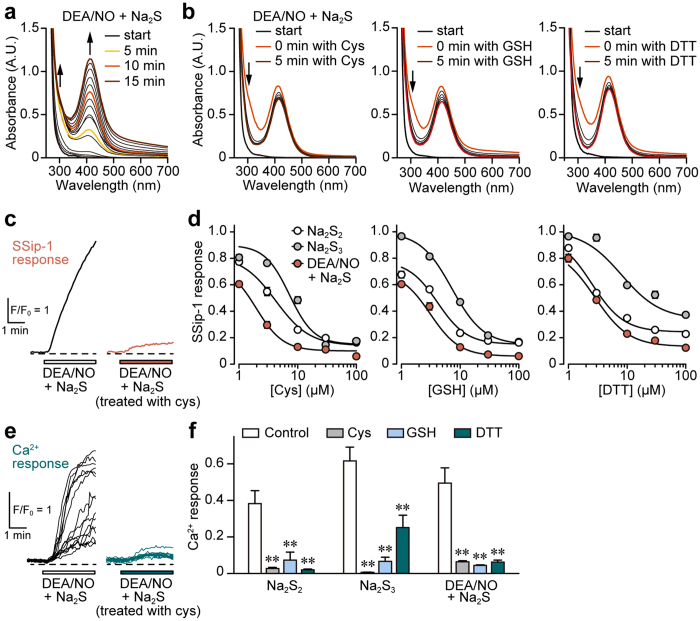
Products from H_2_S and NO are degraded by reducing substances. (**a** and **b**) H_2_S_n_ generated from H_2_S and NO are degraded by cysteine (Cys), GSH, and DTT, while SSNO- is resistant to them. UV-Vis spectra of 5 mM Na_2_S and 2 mM DEA/NO in the presence (**b**) or absence (**a**) of 3 mM each cysteine, GSH, and DTT in 0.5 M HEPES buffer (pH 7.4). Na_2_S and DEA/NO were mixed at the ‘start’, and cysteine, GSH, and DTT were applied 10 min after the ‘start’. H_2_S_n_ is observed at 290–300 nm, and SSNO^−^ at 412 nm. (**c**) SSip-1 responses to the mixture of 20 μM each Na_2_S and DEA/NO pretreated with or without 30 μM cysteine for 5 min. (**d**) Concentration-response relations for degradation of the product from H_2_S and NO by reducing substances. SSip-1 responses to 10 μM Na_2_S_2_, 10 μM Na_2_S_3_, and the mixture of 20 μM each of Na_2_S and DEA/NO with or without pre-treatment with cysteine, GSH, and DTT (n = 28–30). (**e**) Ca^2+^ influx induced by the mixture of H_2_S and NO is suppressed by a pre-treatment with cysteine. Fifteen traces of Ca^2+^-responses to the mixture of 20 μM each of Na_2_S and DEA/NO pretreated with or without 30 μM cysteine were shown. (**f**) The effects of pre-treatment of reducing substances on Ca^2+^ influx induced by the mixture of H_2_S and NO. Pre-treatment of 30 μM each cysteine, GSH and DTT suppressed Ca^2+^-responses to 10 μM Na_2_S_2_, 10 μM Na_2_S_3_, and the mixture of 20 μM each Na_2_S and DEA/NO in AITC-responsive cells (n = 21–35). Na_2_S_2_, Na_2_S_3_, and 5 min pre-incubated mixture of Na_2_S with DEA/NO were treated with cysteine, GSH, and DTT for 5 min. Data are represented as means ± SEM. **P < 0.01 (Dunnett’s test).

SSNO^−^ has also been proposed to be produced by the interaction between H_2_S and NO^15^. SSNO^−^ is resistant to reducing substances, such as cysteine, GSH, and DTT, while H_2_S_n_ is degraded by them^15^,^31^. We examined the generation of H_2_S_n_ and SSNO^−^ from H_2_S and NO, and compared their sensitivity to cysteine, GSH, and DTT by measuring the light absorbance at a specific wavelength. The production of both H_2_S_n_ (absorbance at 290–300 nm) and SSNO^−^ (412 nm) increased after mixing a high concentration 5 mM Na_2_S and 2 mM DEA/NO in a time-dependent manner^15^ (Fig. 6a). In the presence of a high concentration 3 mM each of cysteine, GSH, and DTT, the levels of H_2_S_n_ decreased, while those of SSNO^−^ remained unchanged (Fig. 6b).

The levels of H_2_S_n_ produced from the interaction of physiological concentrations of Na_2_S with DEA/NO in the presence or absence of reducing substances were also examined using SSip-1 in DRG neurons. The levels of H_2_S_n_ increased in DRG neurons on exposure to the mixture of Na_2_S and DEA/NO (20 μM each), while those exposed to cysteine, GSH, and DTT were suppressed in a concentration dependent manner (Fig. 6c and d). A similar result was observed with Na_2_S_2_ and Na_2_S_3_ (Fig. 6c and d).

The effect of H_2_S_n_ generated from H_2_S and NO on the Ca^2+^ influx through the activation of TRPA1 channels was also examined. The mixture of 20 μM each of Na_2_S and DEA/NO induced Ca^2+^ influx, while only a weak Ca^2+^ influx was induced by the mixture exposed to 30 μM each of cysteine, GSH, and DTT (Fig. 6e and f). The effect of 10 μM each of Na_2_S_2_ and Na_2_S_3_ on Ca^2+^ influx was suppressed by these reducing substances in a similar manner (Fig. 6f). The higher concentrations of cysteine were required for suppressing the effect of HNO (Supplemental Fig. 1c). These observations suggest that H_2_S_2_ and H_2_S_3_, rather than SSNO^−^, are produced from the mixture of H_2_S and NO to activate TRPA1 channels.

## Discussion

The present study shows a mechanism of chemical interaction between H_2_S and NO to generate other signaling molecules, H_2_S_2_ and H_2_S_3_, which activate TRPA1 channels (Figs 1 and 2).

The one electron oxidation of sulfide produces thiyl radicals, which readily react with NO radical to generate thionitrous acid (HSNO).


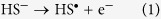



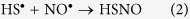


At the physiological pH S^⦁−^ must be the major reactive form and produces polysulfides.


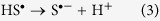



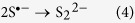



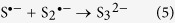


HSNO produces nitroxyl in the presence of H_2_S and persulfide, which in turn reacts with HSNO to produce nitrosopersulfide.


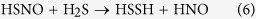



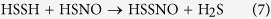


Eberhardt *et al*. reported that HNO and H_2_S_n_ are produced by the interaction of H_2_S with NO^14^. As shown in equations 1, 2 and 6, the interaction of H_2_S and NO generates HSNO, which further reacts with H_2_S to generate HNO^14^,^36^. They measured produced HNO in the presence of 2 μM to 75 μM each of H_2_S and NO by its selective electrode but not characterized the other products H_2_S_n_ (equations 3, 4, 5 and 6) nor the stability of the products against cyanide. HNO is stable to cyanide, while H_2_S_n_ are degraded in cyanolysis. The present study showed that the products from H_2_S and NO are degraded in cyanolysis as readily as H_2_S_n_ (Figs 4 and 5). Although both HNO and H_2_S_n_ can be produced as shown in equation 6, H_2_S_n_ must be the chemical entities that activate TRPA1.

Cortese-Krott *et al*. showed that SSNO^−^ and H_2_S_n_ were produced from H_2_S and NO as shown in equations 2, 6 and 7,^15^,^37^. The study was performed in the presence of 1 to 2 mM NO donors with various ratio of 1 to 10 mM high concentrations of H_2_S. The present study agreed with their results that SSNO^−^ was detected as absorbance at 412 nm in the presence of 5 mM high concentrations of H_2_S with 2 mM NO (Fig. 6). However, it was not detected under physiological concentrations of less than 20 μM of Na_2_S and DEA/NO^15^ (Figs 1a and 6). SSNO^−^, which may have a role as a carrier or donor of NO, relaxed vascular smooth muscle, but they suggested that NO-independent effects must attributed to H_2_S_n_^15^. SSNO^−^ is stable in the presence of high concentration of thiols^15^, while H_2_S_n_ as well as the effective molecules produced from H_2_S and NO are degraded by reducing substances as shown in the present study (Fig. 6). Under physiological conditions the interaction of H_2_S and NO can produce H_2_S_n_ as major products to induce physiological effects.

H_2_S_n_ generation from H_2_S and NO is a fast reaction. The release of NO from DEA/NO is rate limiting for the generation of H_2_S_n_ (Fig. 3), as H_2_S immediately reacts with the released NO to produce H_2_S_n_. This instantaneous production of H_2_S_n_ from H_2_S and NO is important for its physiological roles. H_2_S producing enzymes, 3MST, cystathionine β–synthase, and NOS, are localized to neurons and astrocytes in the central nervous system, and H_2_S and NO interact with each other to produce H_2_S_n_ that activates TRPA1 channels to modify synaptic activity^18^,^23^,^38^,^39^.

In the cardiovascular system, high concentrations of H_2_S are detected in aortic tissues, and the cysteine aminotransferase/3MST, endothelial NOS and cystathionine γ–lyase pathways are localized to vascular endothelium and smooth muscle, respectively^2^,^3^,^40^. H_2_S_n_ produced from H_2_S and NO can activate PKG1α to induce vascular relaxation^27^,^41^.

H_2_S_n_ has various other physiological effects. It regulates the activity of a tumor suppressor PTEN^26^, facilitates the translocation of Nrf2^25^, and suppresses the activity of GAPDH^28^. H_2_S_2_ and H_2_S_3_ are also produced by 3MST from 3MP, and from H_2_S by oxidation or interaction with NO as shown in the present study^20^,^23^,^30^. It is possible that some effects that were previously attributed to H_2_S or NO alone due to H_2_S_n_ generated by the interaction of H_2_S with NO.

Thus interaction of H_2_S and NO yielding H_2_S_n_ plays an important physiological role and provides therapeutic targets for diseases involving these molecules.

## Methods

### Chemicals

All methods were performed in accordance with the guidelines and regulations of chemical substance management and approved by the committees of chemical substance management in the National Institute of Neuroscience, National Center of Neurology and Psychiatry. Diethylamine NONOate (DEA/NO) (Cayman Chemical, Ann Arbor, MI) and Angeli’s salt (Cayman Chemical) were dissolved at 0.1 M in 10 mM NaOH. Na_2_S_2_ (Dojindo, Kumamoto, Japan), Na_2_S_3_ (Dojindo), Na_2_S (Wako pure chemicals, Osaka, Japan), L-cysteine (Cys) (Wako), glutathione (Wako), dithiothreitol (Wako) were dissolved at 0.1 M in ultrapure water. These stock solutions were stored at −80 °C, and were used within a week. NaCN (Wako) was dissolved at 0.5 M in a 0.1 M HEPES buffer (pH 7.4) and stored at −20 °C.

### Isolation of sensory neurons

All the animal procedures were performed in accordance with the guidelines and regulations of animal care and use and approved by the committees of animal care and use in the National Institute of Neuroscience, National Center of Neurology and Psychiatry. DRG were dissected from 1–13 days old Sprague-Dawley rats (CLEA JAPAN, Tokyo, Japan). After the treatment of DRG with collagenase and trypsin, cells were dispersed by pipetting, and suspended in Neurobasal medium supplemented with 2% NS supplement (Wako) and 2% fetal bovine serum, and then seeded onto poly-D-lysine-coated coverslips. The cells, which were incubated in a humidified atmosphere with 5% CO_2_ at 37 °C, were used for imaging experiments within 32 h after seeding.

### Imaging of intracellular Ca^2+^ and H_2_S_n_

Fluo-4 AM (Thermo Fisher Scientific, Waltham, MA, USA) and SSip-1 DA (synthesized as described in ref. 32) were diluted in a HEPES-buffered saline (HBS; in mM: 137 NaCl, 5.4 KCl, 0.8 MgCl_2_, 1.8 CaCl_2_, 10 glucose, 10 HEPES (pH 7.4)). DRG neurons were loaded with 5 μM fluo-4 AM in 0.02% cremophor EL for 45 min at room temperature or with 20 μM SSip-1 DA in 0.02% cremophor EL for 45 min at 37 °C. A coverslip was mounted on an upright microscope (DM LFS, Leica, Heidelberg, Germany) and was perfused with HBS at a rate of 1 ml/min. The recording was started after 15-min perfusion of HBS to let the intracellular probes completely esterized. Fluorescence was recorded every 5 sec with a bandpass filter (excitation at 480/40 nm, emission at 527/30 nm) and a CCD camera (Hamamatsu Photonics, Shizuoka, Japan). Images were acquired using Aquacosmos 2.6 software (Hamamatsu Photonics). Experiments were performed at room temperature.

At the end of the experiments, 50 mM KCl or 30 μM Na_2_S_2_ was applied to neurons to induce maximal Ca^2+^- or SSip-1-responses, respectively. Unless otherwise described, the amplitudes of Ca^2+^- and SSip-1-responses evoked by tested stimulus were normalized by those to KCl and Na_2_S_2_. In Ca^2+^ imaging, neurons responded to AITC with the amplitudes over 20% of those to KCl were considered as TRPA1-expressing neurons.

### LC-MS/MS analysis

Two point five to 5 μl 0.1 M each Na_2_S_2_, Na_2_S_3_, Na_2_S, and DEA/NO stock solutions were diluted in a 95 μl 10 mM HEPES buffer (pH 7.4). Immediately after dilution of Na_2_S_2_ and Na_2_S_3_, or five minutes incubation for the mixture of Na_2_S and DEA/NO), 40 μl of 0.5 M CHES (pH 8.4) and 2 μl 0.05 M monobromobimane were added. Twenty minutes after incubation at room temperature in the dark, samples were neutralized with 15 μl of 30% acetic acid.

Dibimane derivatives were analyzed by the triple-quadrupole mass spectrometer coupled to HPLC (Shimadzu LCMS-8040). Samples were subjected to a reverse phase Symmetry C18 HPLC column (4.6 × 250 mm, Waters) at the flow rate of 0.8 ml/min. The mobile phase consisted of (A) 5 mM ammonium formate in water and (B) 5 mM ammonium formate in a 1:1 solution of water and methanol. Samples were separated by eluting with a gradient: 40% B at 0 min, and 75% B at 8 min and remained it for 10 min. The column oven was maintained at 40 °C. The effluent was subjected to the mass spectrometer using an electrospray ionization (ESI) interface operating in the positive-ion mode. The source temperature was set at 400 °C, and the ion spray voltage at 4.5 kV. Nitrogen was used as a nebulizer and drying gas. The tandem mass spectrometer was tuned in the multiple reaction monitoring mode to monitor mass transitions *m/z* Q1/Q3 432.45/192.00 [S-dibimane + NH_4_]^+^, 464.55/192.00 [SS-dibimane + NH_4_]^+^, 496.60/192.00 [SSS-dibimane + NH_4_]^+^.

### UV-Vis spectrometry

UV-Vis spectra were recorded with a UV/Vis spectrophotometer (Beckman Coulter, Brea, CA, USA). A silica cuvette with a 1.0-cm light path and 100-μl volume was used for the recording. One μl stock solutions of test compounds were diluted in 100 μl solution buffered with 0.5 M HEPES-NaOH (pH 7.4). UV/Vis spectra were recorded immediately as well as every 1 min after the dilution.

### Statistical analysis

All the statistical analysis was performed using OriginLab software (LightStone, Tokyo, Japan). Statistical comparisons between two groups were performed by unpaired Student’s *t*-test. For comparisons between multiple groups, one-way ANOVA was performed followed by Dunnett’s test. *P* < 0.05 was considered statistically significant.

## Additional Information

**How to cite this article**: Miyamoto, R. *et al*. Polysulfides (H_2_S_n_) produced from the interaction of hydrogen sulfide (H_2_S) and nitric oxide (NO) activate TRPA1 channels. *Sci. Rep.* **7**, 45995; doi: 10.1038/srep45995 (2017).

**Publisher's note:** Springer Nature remains neutral with regard to jurisdictional claims in published maps and institutional affiliations.

## Supplementary Material

Supplementary Information
