# Supplementary material for: Polysulfides (H2Sn) produced from the interaction of hydrogen sulfide (H2S) and nitric oxide (NO) activate TRPA1 channels
Source: Sci Rep. 2017 Apr 5;7:45995. doi: 10.1038/srep45995 (PMC5380989; doi:10.1038/srep45995)

Supplementary figures

Polysulfides (H_2S_n) produced from the interaction of hydrogen sulfide (H_2S) and nitric oxide (NO) activate TRPA1 channels

Ryo Miyamoto [1], Shin Koike [2], Yoko Takano [3], Norihiro Shibuya [1], Yuka Kimura [1], Kenjiro Hanaoka [3], Yasuteru Urano [3], Yuki Ogasawara [2], Hideo Kimura [1*]

1. Department of Molecular Pharmacology, National Institute of Neuroscience, National Center of Neurology and Psychiatry, 4-1-1 Ogawahigashi, Kodaira, Tokyo 187-8502, Japan.
2. Department of Analytical Biochemistry, Meiji Pharmaceutical University, 2-552-1 Noshio, Kiyose, Tokyo 204-8588, Japan.
3. Graduate School of Pharmaceutical Sciences, The University of Tokyo, 7-3-1 Hongo, Bunkyo-ku, Tokyo 113-0033, Japan.

*Correspondence to: Dr. Hideo Kimura, Department of Molecular Pharmacology, National Institute of Neuroscience, Kodaira, Tokyo 187-8502, Japan. Tel.:

+81-42-346-1725; Fax: +81-42-346-1755; E-mail: kimura@ncnp.go.jp

Supplementary Fig. 1

Ca^{2+}-responses induced by H_2S_n and Angeli's salt in DRG neurons.

(a) Ca^{2+}-responses visualized by fluo-4 in DRG neurons. Ten micro molar Na_2S_2, 100 μM AITC, and 50 mM KCl were applied to AITC-responsive (+) and unresponsive (−) neurons. (b) The concentration-response relations for Na_2S_2 and Na_2S_3 in AITC-responsive neurons (n = 21–30). (c) The effects of pre-incubation with cysteine on Ca^{2+}-responses to the mixture of Na_2S and DEA/NO and to Angeli's salt (n = 21–34). Cysteine was added to HBS immediately after the dilution of Angeli's salt or 5 min after preparing the mixture of Na_2S with DEA/NO. Data are represented as means ± SEM. $^{**}P < 0.01$ (Dunnett's test).

Supplementary Fig. 2

Detection of H_2S_n with SSip-1 in DRG neurons.

(a) Images of SSip-1-responses after repetitive applications of 10 μM Na_2S_2. Each image was obtained at the time indicated by the corresponding number in (b). (b) Responses of SSip-1 to the repetitive applications of 10 μM Na_2S_2. (c) Bar graphs of responses shown in (b) (n = 29). (d) Traces of SSip-1 concentration-response relations for Na_2S_2. (e) Concentration-response curves for Na_2S_n- or Na_2S_n-induced fluorescence in cells pre-incubated with SSip-1 (n = 30). Responses were normalized by those obtained in response to 30 μM Na_2S_n. Data are represented as means ± SEM.

Supplementary Fig. 3

Specificity of SSip-1 to sulfane sulfur, including H_2S_n, in DRG neurons.

(a) SSip-1 detects H_2S_2, but not HNO. SSip-1 responses to 30 μM Na_2S_2, but not to 1 mM Angeli's salt. (b) Responses of SSip-1 to the mixture of H_2S with NO. SSip-1 responses to the mixture of 20 μM each of Na_2S_2 and DEA/NO, 1 mM Angeli's salt and 1 mM H_2O_2. Compounds were diluted in HBS 5 min before applications to DRG neurons. Responses were normalized to those induced by 30 μM Na_2S_2. (n = 30) (c) The effects of DTT and NaCN on the amplitude of responses to Na_2S_2. Amplitudes of responses before ($\Delta 1$) and after ($\Delta 2$) the application of 10 mM DTT and 20 mM NaCN were defined. (d) High concentrations of DTT and NaCN suppress SSip-1 responses. $\Delta 1/\Delta 2$ values were shown (n = 29-30). Low concentrations of Cys, GSH, and DTT did not affect SSip-1 fluorescence. High concentration of DTT decreased SSip-1 fluorescence probably due to the interference with the sensitive site disulfide bond in SSip-1. Responses were slightly suppressed by NaCN. Data are represented as means ± SEM. $^{**}P < 0.01$ (Dunnett's test).

Supplementary Fig. 1

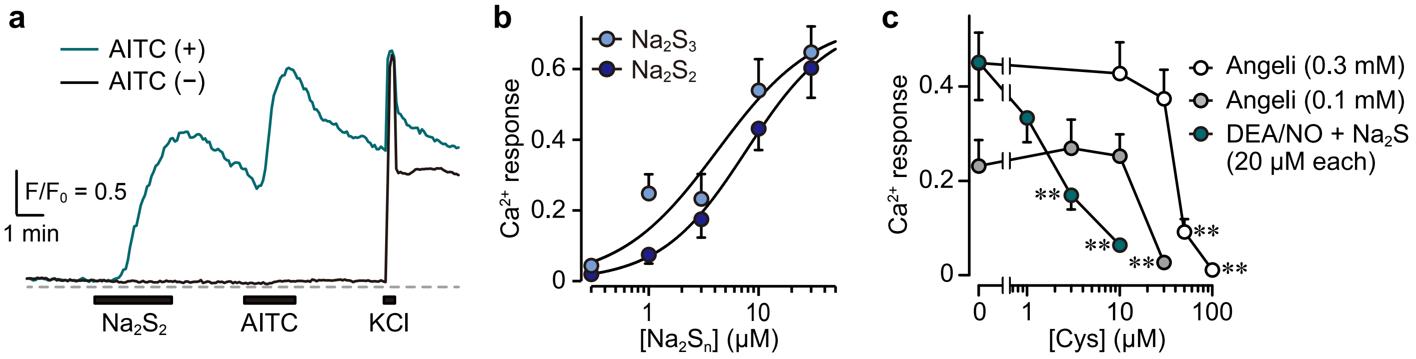

Supplementary Fig. 2

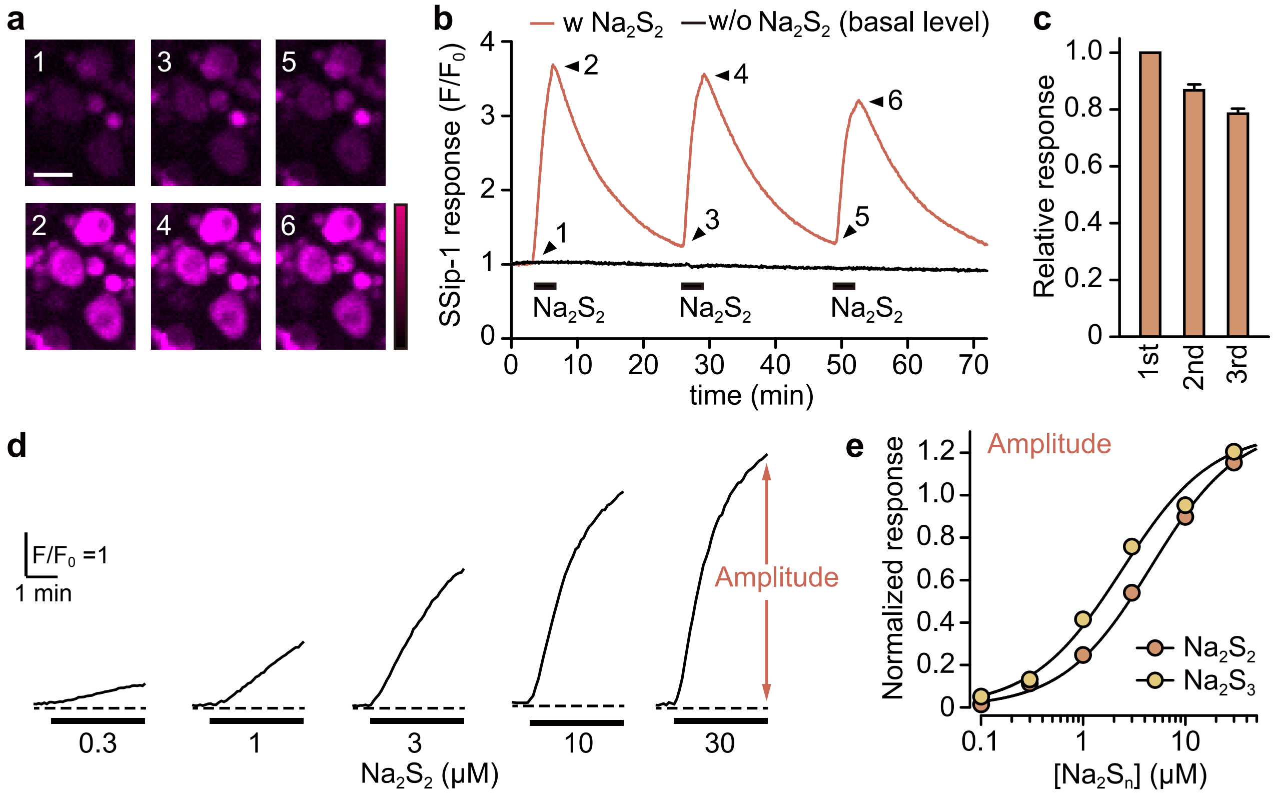

Supplementary Fig. 3

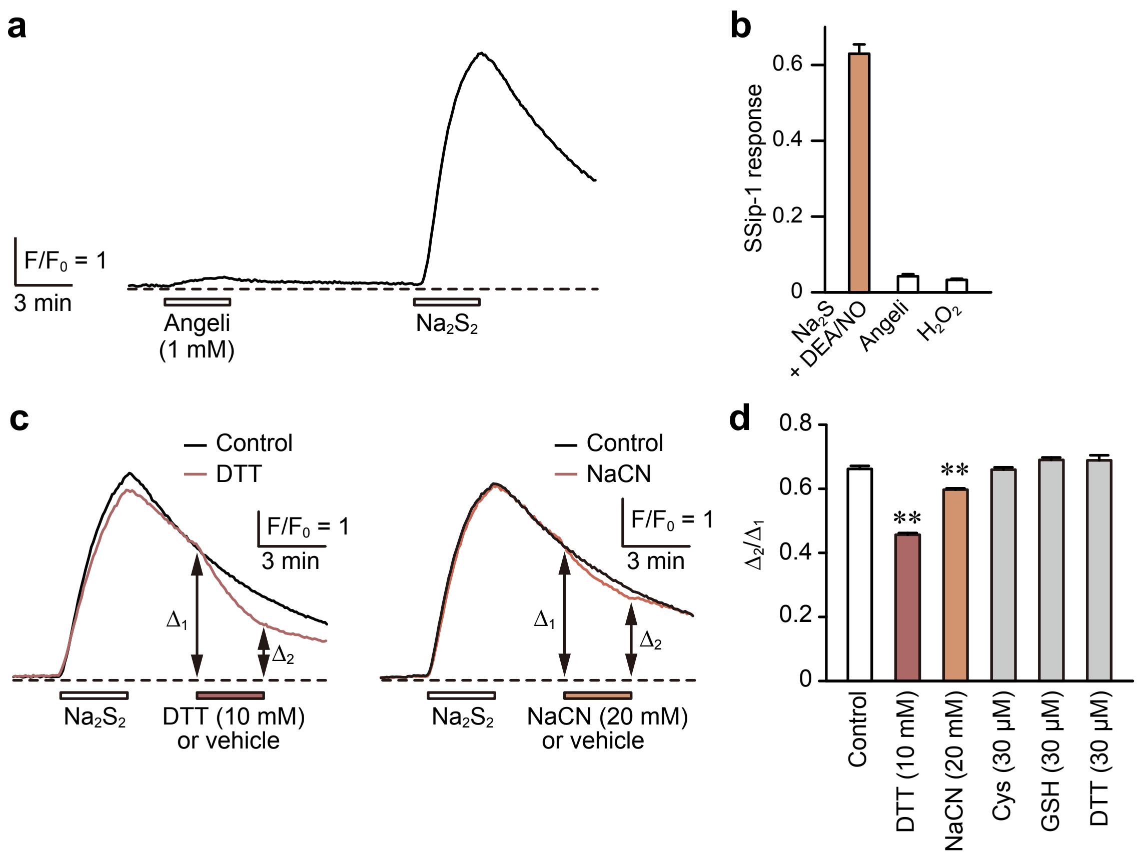

Supplement: Supplementary Information [file srep45995-s1.pdf]
